# Multispectral and thermal infrared data, visual scores for severity of common rust symptoms, and genotypic single nucleotide polymorphism data of three F2-derived biparental doubled-haploid maize populations

**DOI:** 10.1016/j.dib.2024.110300

**Published:** 2024-03-09

**Authors:** Alexander Loladze, Francelino Rodrigues, Cesar D. Petroli, Carlos Muñoz-Zavala, Sergio Naranjo, Felix San Vicente, Bruno Gerard, Osval A. Montesinos-Lopez, Jose Crossa, Johannes W.R. Martini

**Affiliations:** a International Maize and Wheat Improvement Center – CIMMYT, Mexico; b College of Agriculture and Environmental Sciences (CAES), University Mohammed VI Polytechnic (UM6P), Ben Guerir, Morocco; c Facultad de Telemática, Universidad de Colima, Colima, Mexico

**Keywords:** Remote sensing, Unmanned aerial vehicles, UAV, Genome-wide association study, Genomic prediction, Disease resistance, Resistance breeding, High-throughput phenotyping, Corn

## Abstract

Three F2-derived biparental doubled haploid (DH) maize populations were generated for genetic mapping of resistance to common rust. Each of the three populations has the same susceptible parent, but a different resistance donor parent. Population 1 and 3 consist of 320 lines each, population 2 consists of 260 lines. The DH lines were evaluated for their susceptibility to common rust in two years and with two replications in each year. For phenotyping, a visual score (VS) for susceptibility was assigned. Additionally, unmanned aerial vehicle (UAV) derived multispectral and thermal infrared data was recorded and combined in different vegetation indices (“remote sensing”, RS). The DH lines were genotyped with the DarTseq method, to obtain data on single nucleotide polymorphisms (SNPs). After quality control, 9051 markers remained. Missing values were “imputed” by the empirical mean of the marker scores of the respective locus. We used the data for comparison of genome-wide association studies and genomic prediction when based on different phenotyping methods, that is either VS or RS data. The data may be interesting for reuse for instance for benchmarking genomic prediction models, for phytopathological studies addressing common rust, or for specifications of vegetation indices.

Specifications Table

**Table:** 

Subject	Agronomy and Crop Science Genetics: General
Specific subject area	Remote sensing for high-throughput field phenotyping for resistance breeding in maize, here with the example of common rust (CR). Results of genome-wide association studies and genomic prediction were compared when phenotyping was based on human visual scores (VS) to when phenotypes were given by vegetation indices obtained through multispectral and infrared data from images from unmanned aerial vehicles (remote sensing, RS).
Type of data	Tables Raw, filtered, adjusted, imputed
Data collection	1. Visual scores (VS): CR susceptibility was scored per plot by trained staff on a 1 to 9 scale (1=very resistant, 9= very susceptible) 2. Remote sensing (RS) data: Unmanned aerial vehicle eBee Plus (SenseFly Ltd., Cheseaux-Lausanne, Switzerland); Multispectral Parrot Sequoia camera (Parrot Drone SAS, Paris, France) for wavelengths, 550 nm (40 nm full width at half maximum, FWHM), 660 nm (40 nm FWHM), 735 nm (10 nm FWHM), 790 nm (40 nm FWHM); Thermal infrared camera, ThermoMAP (Airinov, Paris, France): 7.5–13.5 µm; Cameras were mounted in separate flights. Pix4D Mapper software (v3.3.24; Pix4D, Lausanne, Switzerland) Vegetation indices (VIs) were calculated per plot from the RS data using the wavelength closest to those of the original definition of the VI. 3. Genotypic data Diversity Array Technology (DArT); Single nucleotide polymorphisms; missing values “imputed by the mean”
Data source location	Remote sensing Multispectral and thermal images acquired from separate successive flights; Flight height approximately 55 m; Flights at midday under sunny conditions; Multispectral camera radiometrically calibrated based on the standard panel provided by the manufacturer; Radiometric adjustment of images based on the incident light sensor of the multispectral camera Institution: International Maize and Wheat Improvement Center (CIMMYT) Town: El Batan, Texcoco de Mora, State of Mexico Country: Mexico
Data accessibility	Repository name: CIMMYT Research Data & Software Repository Network [2] Data identification number: 10548898 Direct URL to data: https://data.cimmyt.org/dataset.xhtml?persistentId=hdl:11529/10548898 Data Use Agreement can be found under “Terms” The access to the genotypic data requires identifying information.
Related research article	A. Loladze, F. A. Rodrigues, C.D. Petroli, C. Munoz, S. Naranjo, F. San Vicente, B. Gerard, O. A. Montesinos-Lopez, J. Crossa, J. W. R. Martini, Use of remote sensing for linkage mapping and genomic prediction for common rust resistance in maize, Field Crops Research, https://doi.org/10.1016/j.fcr.2024.109281[1]

## 1 Value of the Data

The data offers phenotypic data of maize DH lines for different traits related to susceptibility to common rust. The traits comprise visual scores (VS) and remote sensing (RS) traits including vegetation indices. Moreover, the data set provides genotypic data on single nucleotide polymorphisms (SNPs). This combination of phenotypic and genotypic data can for instance be further used for

• benchmarking genomic prediction models with different traits and different types of cross validations for instance related to the population structure,
• benchmarking of models for genome wide association studies (GWAS), for instance models including cofactors or interactions of loci
• for phytopathological studies addressing common rust,
• as a reference data set for high-throughput phenotyping in resistance breeding
• for specifications of vegetation indices

In particular, it may be of value for scientists working in the area of

• high-throughput agricultural phenotyping and breeding,
• statistical geneticists
• phytopathologists

## 2 Background

The objective when generating this data set was to explore the potential of remote sensing (RS) phenotyping methods in the context of resistance breeding, in particular in comparison to low-throughput visual scoring (VS) and when used for follow-up genetic evaluations of the plant material. We compared VS and RS traits with respect to the corresponding results of downstream genome-wide association studies and genomic prediction [1]. The present article describes the data in more detail to provide a solid basis and ideas for a secondary use.

## 3 Data Description

Three different biparental, F2-derived DH populations were generated. All of them had shared the same parent susceptible to common rust. The parent resistant to common rust differed between populations.

The DH lines were genotyped for single nucleotide polymorphisms (SNPs, for more details see Eexperimental Design, Materials and Methods). Genotypic data is available in File Loladze_et_al_genotypes_GID.txt.gz (see Table 1). DHs should be fully homozygous by construction. A heterozygous state of a marker indicates either an error in the genotyping, or in the process of creating the DH line.

**Table 1:** File names, content and data formats of the data set.

File name	File content	Data format	Comment
Loladze_et_al_genotypes_ GID.txt.gz	Imputed genotypic SNP data	Table with allele ID in the first column, location as chromosome and chromosome position in the second and third columns, followed by genotypic information of the different plants. Scale of the data is {−1,0,1}. Rational, non-integer numbers indicate that the data point was missing and needed to be “imputed” by replacing the missing value by the mean of all available data of the respective marker.	Registration is required to access the data; file needs to be extracted
Phenos_pop1_adjusted.txt	Phenotypic data of population 1 for both years, 2019 and 2020, adjusted means across the two replications per genotype and including the block effect.	Genotype identifiers in the first row, adjusted values of different traits in columns	
Phenos_pop2_adjusted.txt	Phenotypic data of population 2 for both years, 2019 and 2020, adjusted means across the two replications per genotype and including the block effect.	Genotype identifiers in the first row, adjusted values of different traits in columns	
Phenos_pop3_adjusted.txt	Phenotypic data of population 3 for both years, 2019 and 2020, adjusted means across the two replications per genotype and including the block effect.	Genotype identifiers in the first row, adjusted values of different traits in columns	
Pop1_2019_raw.txt	Raw (not adjusted) phenotypes for population 1 obtained from the evaluation in 2019	Replication, block, GID and raw trait values in columns for each plot in the field experiment	
Pop1_2020_raw.txt	Raw (not adjusted) phenotypes for population 1 obtained from the evaluation in 2020	Replication, block, GID and raw trait values in columns for each plot in the field experiment	
Pop2_2019_raw.txt	Raw (not adjusted) phenotypes for population 2 obtained from the evaluation in 2019	Replication, block, GID and raw trait values in columns for each plot in the field experiment	
Pop2_2020_raw.txt	Raw (not adjusted) phenotypes for population 2 obtained from the evaluation in 2020	Replication, block, GID and raw trait values in columns for each plot in the field experiment	
Pop3_2019_raw.txt	Raw (not adjusted) phenotypes for population 3 obtained from the evaluation in 2019	Replication, block, GID and raw trait values in columns for each plot in the field experiment	
Pop3_2020_raw.txt	Raw (not adjusted) phenotypes for population 3 obtained from the evaluation in 2020	Replication, block, GID and raw trait values in columns for each plot in the field experiment	

**Fig. 1:**
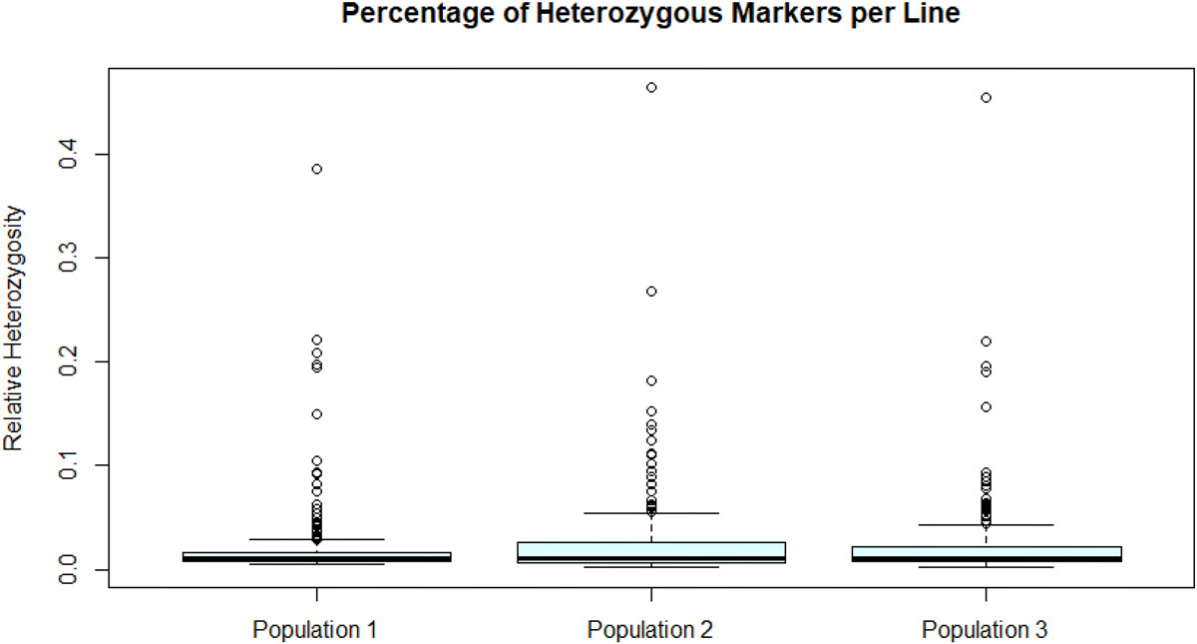
Boxplots of the relative heterozygosity per individual line for the three populations.

Fig. 1 illustrates the distribution of heterozygous calls (the number of “0”s) relative to the total number of calls (sum of the number of “−1”, “0” and “1”s) ***for each individual*** and across the three populations. 15, 23 and 22 individuals show a relative heterozygosity above 5% for populations 1, 2 and 3, respectively.

**Fig. 2:**
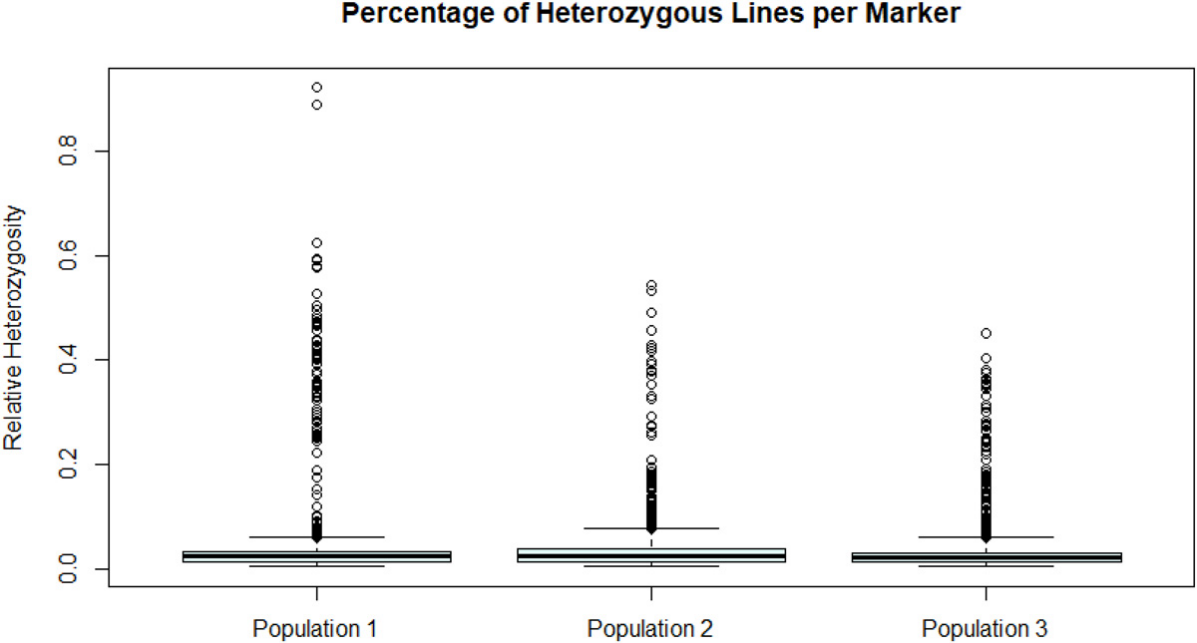
Boxplots of the relative heterozygosity per marker for the three populations.

Fig. 2 illustrates the distribution of heterozygous calls (the number of “0”s) compared to the total number of calls (sum of the number of “−1”, “0” and “1”s) ***for each locus*** and across all lines of the respective population. Out of the 9051 markers, 221, 883 and 316 showed a heterozygosity of higher than 5% for populations 1, 2 and 3, respectively.

The phenotypic raw data is provided by the six files

**Fig. 3:**
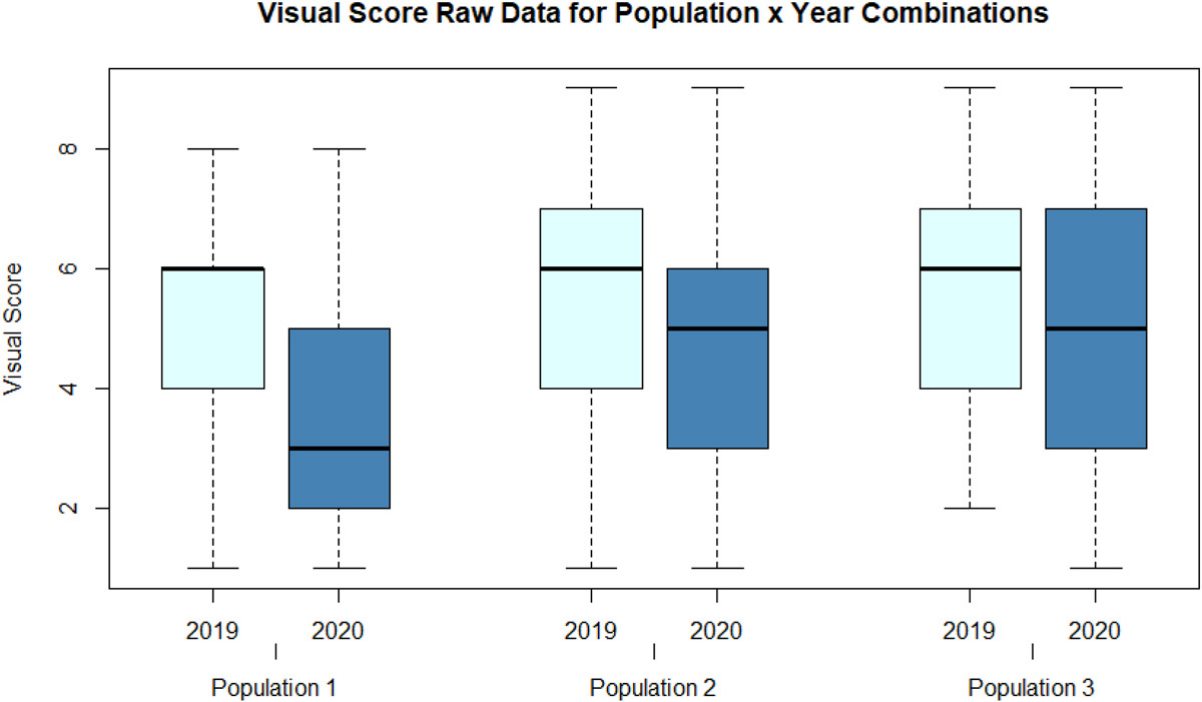
Boxplots of VS raw data across the six combinations of population and year of evaluation.

• Pop1_2019_raw.txt
• Pop1_2020_raw.txt
• Pop2_2019_raw.txt
• Pop2_2020_raw.txt
• Pop3_2019_raw.txt
• Pop3_2020_raw.txt

for the respective combination of population and year (see Table 1). The raw data includes the VS as well as RS traits. For illustrative purposes of the data properties, we highlight the distributions of the VS raw data across population and year in Fig. 3.

The phenotypic adjusted data is provided by the files

• Phenos_pop1_adjusted.txt
• Phenos_pop2_adjusted.txt
• Phenos_pop3_adjusted.txt

**Fig. 4:**
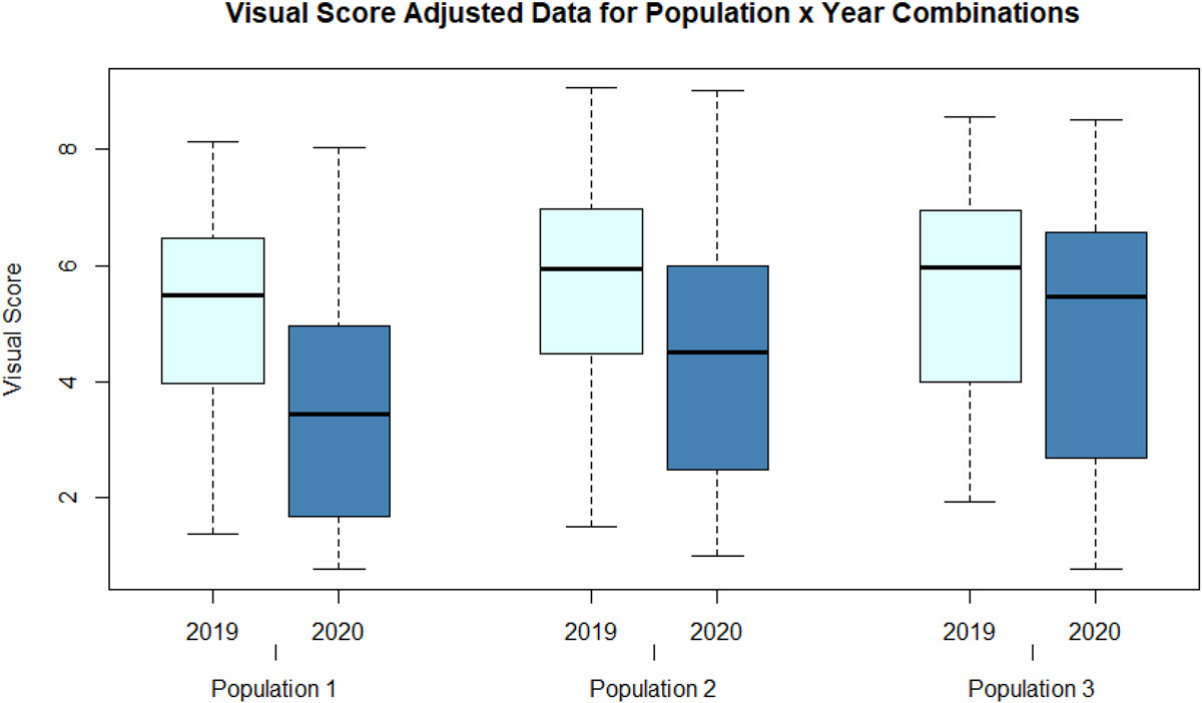
Boxplots of adjusted VS across the six combinations of population and year of evaluation.

Fig. 4 illustrates the distribution of adjusted VS across population and year.

File names, file content, and the data format are described in Table 1.

## 4 Experimental Design, Materials and Methods

1. Creation of plant material:
  • ***Crossing*** of three resistance donors CHWTI23, CHWTI59, and DTMA-17 to susceptible parent DTMA-85
  • ***Growing*** F1 generation at CIMMYT's headquarter El Batan,
  • ***Production of doubled haploid lines*** from 1000 F2 seeds
  • 320 DH lines for population 1 and population 3, 260 DH lines for population 2 for further experiments
2. Two-year field trial evaluation of the three DH populations:
  • ***2 replicates*** (plots) of each genotype
  • Each field plot included ***20 plants*** in 2.5 m long rows with 0.25 m planting distance
  • ***Inoculation*** by spraying a water-Tween 20 suspension of *P. sorghi* urediniospores on two consecutive days
  • ***Field evaluation*** in 2019 and 2020
3. *Visual scoring* of susceptibility to common rust on a 1 to 9 scale by trained personnel (1=very resistant, 9= very susceptible). First scoring was done after the appearance of first symptoms, usually during silking. The second and third evaluation were done in approximately 2 weeks steps after the first evaluation. Only the data of the third time point, that is approximately 4 weeks after the first evaluation was used and is provided in this data set [1].
4. Remote sensing
  • ***Multispectral*** and ***infrared sensors*** on unmanned aerial vehicle eBee Plus (SenseFly Ltd., Cheseaux-Lausanne, Switzerland)
  • Flight height approximately 55 m
  • Flights at midday under sunny conditions
  • ***Parrot Sequoia camera*** (Parrot Drone SAS, Paris, France) recorded the wavelengths: 550 nm (40 nm full width at half maximum, FWHM), 660 nm (40 nm FWHM), 735 nm (10 nm FWHM), 790 nm (40 nm FWHM).
  • Multispectral camera radiometrically calibrated based on the standard panel provided by the manufacturer
  • Radiometric adjustment of images based on the incident light sensor of the multispectral camera
  • ***ThermoMAP*** (7.5–13.5 µm) thermal infrared camera (Airinov, Paris, France) from a separate successive flight.
  • ***Pix4D Mapper software*** (v3.3.24; Pix4D, Lausanne, Switzerland)
  • Remote sensing flights were executed +/-1 day of the time of the corresponding visual scoring
5. Genotypic data
  • provided by Genetic Analyses Service for Agriculture (Spanish acronym SAGA), established at the International Maize and Wheat Improvement Center (CIMMYT), El Batan, Mexico based on the ***Diversity Array Technology*** (DArT)
  • ***Single nucleotide polymorphisms*** were used and markers with more than 40% missing values or minor allele frequency of 0.02 were discarded
  • For the remaining ***9051 markers***, missing values were “***imputed***” by the replacing a missing value by the mean of the respective marker across all genotypes with a marker score.

## Limitations

Generalizability of results obtained from this data set to other traits in the context of high-throughput phenotyping, RS, genomic prediction or genome-wide associations studies will be limited. For the benchmarking of models and methods, the data set provides a specific example of maize and the disease common rust. Results obtained in this context will be specific and the generalizability will be limited.

## CRediT authorship contribution statement

**Alexander Loladze:** Conceptualization, Funding acquisition, Project administration. **Francelino Rodrigues:** Conceptualization, Investigation. **Cesar D. Petroli:** Data curation, Investigation. **Carlos Muñoz-Zavala:** Data curation, Investigation. **Sergio Naranjo:** Resources, Investigation. **Felix San Vicente:** Resources, Investigation. **Bruno Gerard:** Project administration, Supervision. **Osval A. Montesinos-Lopez:** Conceptualization. **Jose Crossa:** Conceptualization. **Johannes W.R. Martini:** Conceptualization, Data curation, Formal analysis, Writing – original draft, Writing – review & editing.

## Data Availability

Replication Data for: Use of Remote Sensing for Genome-Wide Association Studies and Genomic Prediction (Original data) (Dataverse) [2].

## Appendix Supplementary materials

Image, application 1
